# Rise and shine for eating right: the link between healthy nutrition and chronotype among young adults

**DOI:** 10.3389/fnut.2023.1285015

**Published:** 2023-10-12

**Authors:** Hande Mortaş, Büşra Ayhan, Semra Navruz Varlı, Süleyman Köse, Duygu Ağagündüz, Saniye Bilici

**Affiliations:** ^1^Department of Nutrition and Dietetics, Faculty of Health Sciences, Gazi University, Ankara, Türkiye; ^2^Department of Nutrition and Dietetics, Faculty of Health Sciences, Artvin Çoruh University, Artvin, Türkiye

**Keywords:** chronotype, dietary intake, eating habits, healthy nutrition, macronutrient intake

## Abstract

**Aim:**

Many studies have examined the relationship between chronotype and general health. Because healthy eating is the basis of health aim of this study is to evaluate the relationship between healthy nutrition attitudes and chronotype in young adults. Methods: This cross-sectional study was conducted with 1,400 young adults (936 females and 464 males). Chronotype was assessed by the Turkish version of the Morningness-Eveningness Questionnaire (MEQ) and the Attitude Scale for Healthy Nutrition (ASHN) was applied by face-to-face interview. Furthermore, 24-h dietary recall was collected. The scores of nutrient adequacy ratio (NAR) and mean adequacy ratio (MAR) were calculated. The anthropometric measurements including body weight and height were taken.

**Results:**

The chronotype distribution of participants according to the MEQ score and MAR score were not significantly different between females and males (*p* > 0.05). The percentage of participants with high healthy eating attitude was lowest in the evening chronotype and highest in the morning chronotype (49.7, 37.7 and 25.4% in morning-, intermediate-, evening-types, respectively; *p* = 0.000 for all groups according to the reciprocal comparisons). There was a positive correlation of the MEQ scores with the scores of ASHN (*r* = 0.282, *p* < 0.01). ASHN score was shown to be a predictor for MEQ score (*β* = 0.280, *p* = 0.000). Moreover, the score of “poor eating habits” was shown to be highest in the evening chronotype and lowest in the morning chronotype (14.9 ± 3.0 and 13.1 ± 3.0, respectively; *p* < 0.01).

**Conclusion:**

The results of this study indicate that individuals with the evening chronotype are more deficient in terms of healthy nutrition attitudes. Further studies with larger populations will potentially guide the development of health promotion strategies to prevent and treat chronic diseases based on an individual’s chronotype.

## 1. Introduction

Today, chronobiology has started to be one of the fields of science that can lead to results associated with many diseases such as cardiovascular diseases, obesity, and mood disorders (1–3). Desynchronization due to various factors, including jet-lag, genetic variations, and night shift working in the circadian system, which gives rhythms to physiological functions synchronized with cyclical environmental signals, increases the risk of disease (4). This desynchronization state is called circadian disruption or chronodisruption (5). Circadian expression varies among people due to age, race, sex, genetic, and/or environmental factors (6). Due to these factors, individuals either consciously choose the rest/activity times or the rest/activity times occur because of the phase entrainment that appears under the influence of factors causing chronodisruption. The term describing these individual differences in rest/active times is chronotype (7). According to their chronotypes, individuals are generally classified as morning, intermediate or evening chronotype. Morning individuals tend to sleep early and perform their mental performance and physical activity early in the day. In contrast, evening individuals, on the contrary, prefer late hours of the day to sleep and for all these activities. It has been reported that more than half of the individuals have the intermediate chronotype apart from these two chronotypes (6). Individuals with the evening chronotype are shown to be prone to several health problems, including metabolic syndrome, mood disorders, emotional and behavioral problems (4, 7–10). In addition, it has been demonstrated that the increase in food consumption and unhealthy eating behaviors observed in individuals with the evening chronotype are so high compared to individuals with morning chronotype, thus contributing to the increase in the risk of obesity and metabolic syndrome development in individuals in the evening chronotype (8, 11–14). In another study by Rosi et al. (15), morning individuals have been shown to consume sugar-containing foods and ultra-processed oils at significantly lower levels. In a systematic review that included a total of 39 observational studies with a total of 377,797 participants, it was found that evening individuals had higher blood glucose, glycated hemoglobin, LDL cholesterol, and triglycerides levels and risks of diabetes, cancer, and depression than morning individuals, and there was no difference between the two groups in dietary energy intake (16). In recent studies, individuals with the evening chronotype have been found to have lower intakes of milk products, vegetables-fruits, dietary fiber and generally show unhealthy eating behaviors and attitudes (17–19). In another study investigating the relationship between adherence to the Mediterranean diet, which is one of the most ideal diets for healthy nutrition, and chronotype, morning individuals’ adherence to the Mediterranean diet was higher than evening and intermediate individuals (20).

As summarized above, the effect of the chronotype formed by exogenous or endogenous factors on nutrition is undeniable. However, little is known about the association of chronotype with healthy nutrition attitude in young adults, whose nutritional choices start to change due to reasons such as starting to work, staying at home apart from the family, starting university, and social interaction with individuals with different food preferences (12). Therefore, this study aims to investigate the association of chronotype with healthy nutrition attitudes in young adults.

## 2. Methods

### 2.1. Participants and study design

The participants in this cross-sectional study as volunteers were 1,400 healthy young adults (936 females and 464 males), aged 19–30 years. The number of samples was determined using G*power 3 program. As a result of the analysis performed by taking alpha (*α*) = 0.05, power (1–*β*) = 0.95, it was calculated that at least 1,220 individuals should be included in the study. Exclusion criteria for participation in the study were: following any diet or using medication and sleeping pills due to chronic diseases or voluntarily, being pregnant or in the period of lactation, leaving any of the scale questions, and having any history of medically diagnosed sleep disorders. Additionally, those who had been doing regular physical activity for the last week, those who were physically active due to the department they studied at university, and those who were physically active at work were not included in the study.

This study was completed following the 1964 Helsinki declaration. The research protocol of the study was approved by the Ethics Committee of the Gazi University of Ankara/Turkey (Approval No. 2021–1041), and written informed consent was obtained from all volunteers.

### 2.2. Study protocol

The questionnaire covered general characteristics like participants’ age, current dietary supplement use, body weight (kg), and height (cm) based on self- declaration. Chronotype was assessed by the Turkish version of the Morningness-Eveningness Questionnaire (MEQ), and the Attitude Scale for Healthy Nutrition (ASHN) was applied to the participants by the researchers *via* face-to-face interviews. Moreover, information on dietary intake was collected using 24-h dietary recall.

### 2.3. Anthropometric measurements

The body weight (kg) and height (cm) taken by the researchers were used to calculate body mass index (BMI) and evaluated according to the World Health Organization classification (21).

### 2.4. Morningness-Eveningness Questionnaire (MEQ)

The original questionnaire, which is used as the “Morningness-Eveningness Questionnaire (MEQ)” in the literature, which subjectively determines the circadian type, was developed by Horne et al. (22). This scale is a Likert-type scale consisting of nineteen questions. The MEQ’s confidence coefficient (Cronbach’s alpha), performed validity and reliability of its Turkish version by Pündük et al. (23), was calculated as 0.834 in the present study. In the analyses, the MEQ score was used either as a continuous variable or as a categorical variable by dividing the MEQ score into thirds based on score tertiles based on the scores in the present study, as morning (56–78 points), intermediate (44–56 points), and evening (0–44 points) chronotypes.

### 2.5. Attitude Scale for Healthy Nutrition (ASHN)

Attitude Scale for Healthy Nutrition was developed by Demir-Tekkurşun and Cicioğlu (24). The scale is a Likert-type scale consisting of 21 questions. Participants with 21 points on the ASHN have very low, 23–42 points low, 43–63 points average, 64–84 points high, and 85–105 points ideally healthy eating attitude. Also, these questions referred to 4 factors consisting “nutrition knowledge,” “emotion for nutrition,” “positive nutrition habits” and “poor eating habits.” In the analyses, the ASHN score was used either as a continuous variable or as a categorical variable, as described above. In this study, no participant was found in the category of “ideally healthy eating attitude” according to the results. The confidence coefficient (Cronbach’s alpha) of the ASHN was calculated as 0.825.

### 2.6. Assessment of dietary intake

Evaluation of diet composition and nutritional adequacy of the diet was performed using 24-h dietary recall. The energy, macronutrients, and micronutrients were analyzed using the BeBiS (Nutrition Information System) program (version 8.2). Nutrient adequacy ratio (NAR) scores were calculated by comparing individual daily consumption of nutrients with Dietary Reference Intake (DRI) levels categorized by age and gender (25). The percentage of young adults’ dietary protein intake meeting the DRI requirement was calculated as 0.8 g/kg body weight for the reference body weight, as specified in the DRI (25). In this study, NAR scores were calculated as percentages for a total of 12 nutrients, including carbohydrates, protein, fiber, calcium, potassium, zinc, iron, vitamin A, B_6_, B_12_, folate, and vitamin C (26). The mean adequacy ratio (MAR) score was obtained as a percentage by averaging the NAR scores for 12 nutrients (26).

### 2.7. Statistical analysis

All statistical data were analyzed using SPSS (The Statistical Package for Social Sciences) Version 22.0 (SPSS Inc., Chicago, IL, USA). Frequencies and ± standard deviation (SD) were calculated to measure central tendency and propagation, and *p* < 0.05 was significant using two-tailed tests. Student’s t-test and one-way ANOVA were used to show statistical differences between the mean values. Chi-square test was performed to compare characteristic features. The bivariate correlation of MEQ with age, BMI, ASHN, energy intake, NAR, and MAR was determined using Pearson correlation adjusted for BMI and age. Multiple linear regression testing was performed using gender, age, BMI, and the scores of ASHN and MAR as predictors of the MEQ score. This regression model aims to identify the parameters that act together by describing the relationship between the markers and investigate the interchangeability of the commonly applied measurements. As a result, the regression model created with gender, age, BMI, ASHN and MAR scores, which represent healthy nutrition, revealed that the ASHN score can be considered a good model for predicting the individual’s chronotype.

## 3. Results

General characteristics of the participants, including age, BMI, current dietary supplement use, MEQ score, dietary energy intake, and NAR score according to gender, are presented in Table 1. Based on the results from BMI, there were 6.9 and 35.7% underweight; 65.7 and 53.2% normal weight; 27.4 and 11.1% overweight and obese participants in males and females, respectively (*p* = 0.000 for all BMI groups). Morning-, intermediate-, and evening-type chronotypes were 23.0, 48.6, and 28.4% of the total participants, respectively. The chronotypes did not differ significantly in intermediate- and evening-types except morning-type, based on gender. The percentage of females with morning-type chronotype was significantly higher than that of males (24.1 and 20.7%, respectively; *p* < 0.01). The chronotype distributions of participants according to the MEQ score tertile and MAR score were not significantly different between females and males. The percentage of those with a high healthy nutrition attitude was found to be higher in males than in females (40.2 and 30.6%, respectively; *p* = 0.000). According to ASHN factor categories, it was found that although females had a higher level of nutrition knowledge than males (*p* = 0.014), they showed a higher tendency toward poor nutrition, and their emotions affected the nutritional status more (*p* > 0.05 in both categories). While there was no significant difference between the MAR scores of participants according to their gender (*p* > 0.05), NAR scores of protein, carbohydrate, fiber, zinc and vitamin C were found to be higher in females; potassium, iron, vitamin B_6_ and folate NAR scores were shown to be higher in males (*p* < 0.05 for all these parameters between males and females).

**Table 1:** General characteristics and the score of the morningness–eveningness questionnaire and attitude scale for healthy nutrition according to gender.

General characteristics	All participants (*n* = 1,400)	Females (*n* = 936)	Males (*n* = 464)
Age (y) [ $\bar{x}$ ± SD]	21.1 ± 2.0	21.0 ± 1.8	21.1 ± 2.4
		*t* = 0.480 *p* = 0.632	
Weight (kg) [ $\bar{x}$ ± SD]	63.3 ± 12.3	57.7 ± 9.1	74.6 ± 11.4
		*t* = 29.939 *p* = 0.000**	
Height (cm) [ $\bar{x}$ ± SD]	168.1 ± 8.7	163.7 ± 5.6	176.9 ± 6.8
		*t* = 38.826 *p* = 0.000**	
BMI (kg/m^2^) [*n* (%)]			
Underweight	366 (26.1)	334 (35.7)^a^	32 (6.9)^b^
Normal weight	803 (57.4)	498 (53.2)^a^	305 (65.7)^b^
Overweight and obese	231 (16.5)	104 (11.1)^a^	127 (27.4)^b^
		*χ^2^* = 156.529 *p* = 0.000**	
Current supplement use [*n* (%)]	160 (11.4)	112 (12.0)	48 (10.4)
		*χ^2^* = 2.819 *p* = 0.244	
MEQ score [*n* (%)]			
Morning type	322 (23.0)	226 (24.1)^a^	96 (20.7)^b^
Intermediate	681 (48.6)	462 (49.4)^a^	219 (47.2)^a^
Evening type	397 (28.4)	248 (26.5)^a^	149 (32.1)^a^
		*χ^2^* = 5.359 *p* = 0.069	
ASHN score [*n* (%)]			
Low	17 (1.2)	12 (2.6)^a^	5 (0.5)^b^
Middle	865 (61.8)	310 (66.8)^a^	555 (59.3)^b^
High	518837.0)	142 (30.6)^a^	376 (40.2)^b^
		*χ^2^* = 21.268 *p* = 0.000**	
ASHN factor categories [ $\bar{x}$ ± SD]			
Nutrition knowledge	17.4 ± 2.9	17.7 ± 2.8^a^	16.9 ± 3.1^b^
		*t* = 5.058 *p* = 0.014*	
Emotion for nutrition	13.5 ± 3.4	13.8 ± 3.3^a^	13.0 ± 3.4^a^
		*t* = 3.703 *p* = 0.242	
Positive nutrition habits	15.7 ± 2.8	15.7 ± 2.8^a^	15.8 ± 2.7^a^
		*t* = 1.068 *p* = 0.728	
Poor eating habits	14.0 ± 3.0	14.3 ± 2.9^a^	13.6 ± 3.1^a^
		*t* = 3.899 *p* = 0.054	
Dietary intake [ $\bar{x}$ ± SD]			
Energy	1699.4 ± 565.1	1627.9 ± 510.8	1843.6 ± 638.0
		*t* = 6.832 *p* = 0.000**	
NAR score [ $\bar{x}$ ± SD]			
NAR protein	93.9 ± 14.0	94.6 ± 12.6	92.3 ± 16.3
		*t* = −3.000 *p* = 0.003**	
NAR carbohydrate	88.7 ± 19.5	89.5 ± 18.2	87.1 ± 21.8
		*t* = −2.191 *p* = 0.029*	
NAR fiber	60.9 ± 23.9	66.7 ± 23.6	49.1 ± 20.1
		*t* = −13.809 *p* = 0.000**	
NAR calcium	56.7 ± 25.0	55.8 ± 24.1	58.5 ± 26.7
		*t* = 1.957 *p* = 0.051	
NAR potassium	44.2 ± 17.6	43.6 ± 16.8	45.6 ± 18.9
		*t* = 2.063 *p* = 0.039*	
NAR zinc	83.7 ± 20.6	86.4 ± 19.1	78.3 ± 22.5
		*t* = −7.126 *p* = 0.000**	
NAR iron	64.1 ± 26.7	50.8 ± 19.8	91.1 ± 16.6
		*t* = 37.656 *p* = 0.000**	
NAR vitamin A	73.0 ± 27.2	76.4 ± 25.8	66.2 ± 28.5
		*t* = −6.701 *p* = 0.000**	
NAR vitamin B_6_	76.9 ± 23.0	76.0 ± 22.7	78.8 ± 23.7
		*t* = 2.136 *p* = 0.033*	
NAR vitamin B_12_	87.7 ± 23.9	87.1 ± 24.1	88.9 ± 23.3
		*t* = 1.408 *p* = 0.159	
NAR folate	56.6 ± 25.6	54.9 ± 25.1	59.7 ± 26.3
		*t* = 3.268 *p* = 0.001**	
NAR vitamin C	72.1 ± 32.1	76.7 ± 30.7	65.0 ± 33.7
		*t* = −5.898 *p* = 0.000**	
MAR [ $\bar{x}$ ± SD]	71.5 ± 14.9	71.5 ± 14.4	71.7 ± 15.7
		*t* = 0.309 *p* = 0.758	

All percentages are calculated on columns. ^a–c^ Represent the statistically significant differences among the line groups at *p* < 0.05. ASHN, Attitude Scale for Healthy Nutrition; BMI, Body Mass Index; MEQ, Morningness-Eveningness Questionnaire; NAR, Nutrient Adequacy Ratio; MAR, Mean Adequacy Ratio. *p < 0.05; **p < 0.001.

Descriptive statistics of BMI, energy intake, scores of ASHN, NAR, and MAR according to chronotype are shown in Table 2. The participants with low healthy nutrition attitudes were more in the evening group than those in morning and intermediate groups. It was also presented that as the transition from morning chronotype to intermediate and night chronotype, respectively, the percentages of participants with high healthy nutrition attitude decreased significantly (49.7, 37.7 and 25.4%, respectively; *p* = 0.000 for all groups according to the reciprocal comparisons). In addition, it was determined that positive nutrition habits were highest in the morning chronotype group and lowest in the evening chronotype group (*p* < 0.01). Similarly, poor eating habits were shown to be highest in the evening chronotype and lowest in the morning chronotype (p < 0.01). Moreover, it has been revealed that nutritional knowledge levels did not differ significantly between individuals with morning chronotype and those with intermediate and evening chronotypes (*p* < 0.05). “Emotion for nutrition” was found to be highest in morning chronotype and lowest in evening chronotype (*p* < 0.01). Participants with the evening-type chronotype were indicated to have higher NAR carbohydrate, iron, and vitamin C scores than those with morning- and intermediate-type chronotypes (*p* > 0.05). The mean NAR scores, including fiber, calcium, potassium, zinc, vitamin A, vitamin B_12_, and folate among morning-types, were higher than those among intermediate- and evening-types. However, these differences were found to be statistically significant only in vitamin A and calcium NAR scores (*p* < 0.01 in both scores).

**Table 2:** Descriptive statistics of the BMI, energy intake, the scores of ASHN, NAR, and MAR according to choronotype.

	Morning type	Intermediate	Evening	*p* values
BMI (kg/m^2^) [ $\bar{x}$ ± SD]	22.3 ± 3.1	22.1 ± 3.4	22.4 ± 3.5	0.450
Energy intake (kcal) [ $\bar{x}$ ± SD]	1718.0 ± 564.4	1684.8 ± 567.2	1709.3 ± 562.9	0.629
ASHN score [n (%)]				
Low	3 (0.9)^a,b^	2 (0.3)^b^	12 (3.0)^a^	0.000**
Middle	159 (49.4)^a^	422 (62.0)^b^	284 (71.5)^c^	
High	160 (49.7)^a^	257 (37.7)^b^	101 (25.4)^c^	
ASHN factor categories [ $\bar{x}$ ± SD]				
Nutrition knowledge	17.5 ± 3.2^a,b^	17.6 ± 2.7^a^	17.1 ± 3.2^b^	0.030*
Emotion for nutrition	14.2 ± 3.5^a^	13.6 ± 3.3^b^	12.8 ± 3.2^c^	0.000**
Positive nutrition habits	16.6 ± 2.5^a^	15.6 ± 2.7^b^	15.2 ± 2.8^c^	0.000**
Poor eating habits	13.1 ± 3.0^a^	14.1 ± 2.8^b^	14.9 ± 3.0^c^	0.000**
NAR score [ $\bar{x}$ ± SD]				
NAR protein	93.9 ± 14.0	93.8 ± 14.1	93.8 ± 13.9	0.993
NAR carbohydrate	88.3 ± 19.6	87.9 ± 20.1	90.3 ± 18.3	0.156
NAR fiber	62.6 ± 23.0	60.3 ± 24.5	60.6 ± 23.8	0.339
NAR calcium	60.9 ± 24.3^a^	54.9 ± 25.6^b^	56.2 ± 24.3^b,c^	0.002**
NAR potassium	45.0 ± 17.0	43.5 ± 17.6	44.8 ± 24.3	0.310
NAR zinc	84.9 ± 20.3	83.7 ± 20.9	82.7 ± 20.3	0.362
NAR iron	63.8 ± 26.6	63.2 ± 26.1	65.9 ± 27.9	0.269
NAR vitamin A	77.3 ± 25.3^a^	72.3 ± 27.4^b^	70.8 ± 27.8^b,c^	0.004**
NAR vitamin B_6_	77.6 ± 22.8	75.9 ± 23.3	78.1 ± 22.7	0.286
NAR vitamin B_12_	88.9 ± 21.7	87.2 ± 24.7	87.3 ± 24.1	0.559
NAR folate	59.3 ± 25.8	55.6 ± 25.1	56.0 ± 26.2	0.089
NAR vitamin C	72.2 ± 32.3	71.3 ± 32.5	73.4 ± 31.3	0.583
MAR [ $\bar{x}$ ± SD]	72.9 ± 14.1	70.8 ± 15.2	71.7 ± 14.9	0.113

All percentages are calculated on columns. ^a,b^ Represent the statistically significant differences among the line groups at *p* < 0.05. ASHN, Attitude Scale for Healthy Nutrition; BMI, Body Mass Index; MEQ, Morningness-Eveningness Questionnaire; NAR, Nutrient Adequacy Ratio; MAR, Mean Adequacy Ratio. *p < 0.05; **p < 0.001.

Table 3 presents the adjusted correlation for the BMI and age of MEQ score with ASHN, energy intake, NAR, and MAR. In general, there were positive correlations of the MEQ score with the scores of ASHN and NAR vitamin A (*r*_s_ = 0.282 and 0.076, respectively; *p* < 0.01).

**Table 3:** Correlation of the MEQ with ASHN, energy intake, NAR, and MAR.

	MEQ	
Variables	*r*	*p*
ASHN score	0.282	0.000**
Energy intake	−0.019	0.479
NAR score		
NAR protein	0.002	0.998
NAR carbohydrate	−0.039	0.142
NAR fiber	0.048	0.075
NAR calcium	0.049	0.070
NAR potassium	−0.003	0.9408
NAR zinc	0.052	0.051
NAR iron	−0.041	0.122
NAR vitamin A	0.076	0.004**
NAR vitamin B_6_	−0.002	0.942
NAR vitamin B_12_	0.020	0.464
NAR folate	0.029	0.2674
NAR vitamin C	−0.014	0.613
MAR	0.024	0.363

ASHN, Attitude Scale for Healthy Nutrition; MEQ, Morningness-Eveningness Questionnaire; NAR, Nutrient Adequacy Ratio; MAR, Mean Adequacy Ratio.**p < 0.001, adjusted age and body mass index.

Multiple regression analyses using gender, age, BMI, and ASHN and MAR scores as predictors of the MEQ score are summarized in Table 4. ASHN score was shown to be a predictor for MEQ score (*β* = 0.280, *p* = 0.000). According to the regression analysis results, a one-unit increase in the ASHN score caused a 0.343-unit increase in the MEQ score, indicating an increase in the tendency to be a morning individual.

**Table 4:** Multiple regression analyses using the gender, age, BMI, and the scores of ASHN and MAR, as predictors of the MEQ score.

					95%CI	
Variables	*Β* (SE)	Beta	*t*	*p*	Lower	Upper
Gender	0.972 (0.562)	0.047	1.729	0.084	−0.131	2.074
Age	0.018 (0.125)	0.004	0.147	0.883	−0.227	0.263
BMI	0.060 (0.080)	0.020	0.749	0.454	−0.097	0.216
ASHN score	0.343 (0.032)	0.280	10.691	0.000**	0.280	0.406
MAR score	−0.010 (0.017)	−0.016	−0.598	0.550	−0.044	0.023

ASHN, Attitude Scale for Healthy Nutrition; BMI, Body Mass Index; MAR, Mean Adequacy Ratio. Constant Morningness-Eveningness Questionnaire score, Predictors gender, age, BMI, ASHN score, MAR score. **p < 0.001.

**Figure 1:**
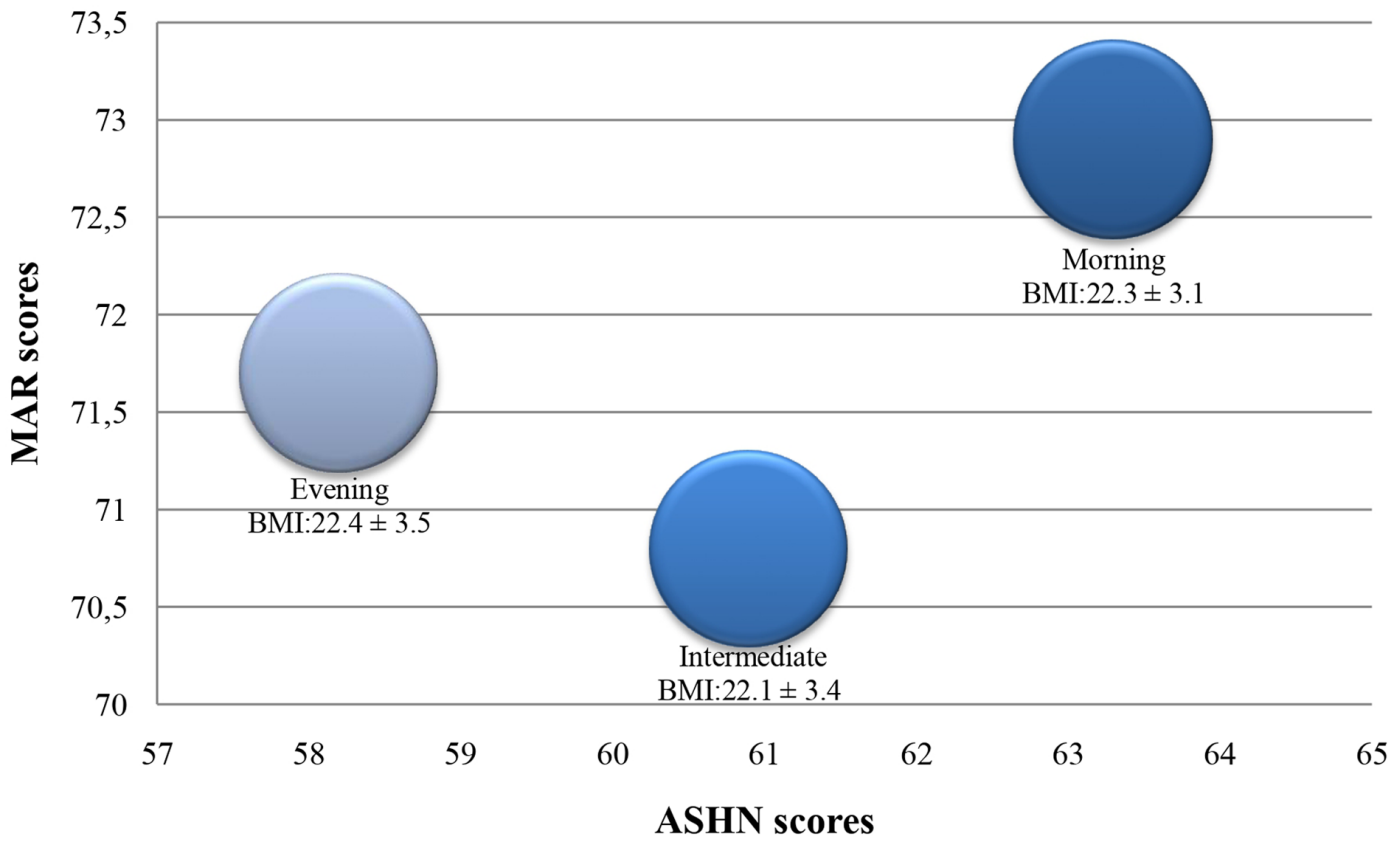
Healthy nutritional status of individuals according to their chronotypes. ASHN, Attitude Scale for Healthy Nutrition; BMI, Body Mass Index; MAR, Mean Adequacy Ratio.

Figure 1, which allows to visually summarize the study’s main results, visualizes the healthy nutritional status and BMI of individuals according to their chronotypes. The colors of the bubbles in the figure represent different chronotypes. Dark blue represents morning individuals, light blue represents evening individuals, and blue represents intermediate individuals. The diameter of the bubbles reflects the mean BMI of the individual in each chronotype group. Since there was no statistically significant difference between the BMIs of individuals according to chronotypes (Table 2), the bubble sizes were also similar (Figure 1). Individuals’ ASHN scores are seen on the x-axis of the figure. It was shown that morning individuals had the highest ASHN scores and evening individuals had the lowest ASHN scores. Although there was no statistically significant difference between the MAR scores of individuals (Table 2), individuals were found to have the highest MAR score in the morning (Figure 1).

## 4. Discussion

Individuals with the morning chronotype tend to have an earlier sleep–wake schedule and prefer to be active and eat in the morning. In contrast, individuals with the evening chronotype generally sleep at a later time and function best in the late afternoon or in the evening (27). Increasing evidence supports the argument that there is a relationship between chronotype and eating attitudes. In this study, the relationship between healthy nutrition attitudes and chronotype among young adults has been supported.

Previous meta-analyses of data synthesized from studies of adolescents and adults indicated that males were more evening-oriented than females (28, 29). The intermediate type refers to individuals who can be either morning or evening. In this study, although most participants (49.4 and 47.2%, respectively in females and males) were intermediate-type, it was found that 26.5 and 32.1% of females and males were evening-type, respectively. However, the difference in chronotype distribution of participants was significant only among morning-types according to gender (24.1 and 20.7% in females and males, respectively, *p* < 0.05). One of the factors that can cause chronotype differences between individuals is gender. The biological rhythms of males and females are quite different. Similar to the present study, it was known that females generally tend to go to bed earlier, wake up earlier, and prefer morning activities (30, 31).

Eating habits determine daily rhythms. The timing of dietary intake, called chrononutrition, is a factor that an individual’s chronotype can influence. Studies showed that individuals in the evening chronotype eat fewer meals, eat more at meals, and are prone to delay eating because they wake up later (32, 33). The eating behavior exhibited in the evening chronotype negatively affected the healthy eating behavior similar to this study (34–36). One of the study’s important outputs was the determination of a higher score in the “poor eating habits” category in the evening chronotype group than in the intermediate and morning chronotype groups. In addition, ASHN was found to be a predictor for the MEQ score in the study. According to this finding, the healthy eating attitude of individuals is an important determinant of their tendency to be with morning chronotype. Similarly, in a study conducted with school-aged children, evening-oriented children were shown to be at risk of consuming fast food (27). Moreover, Mazri et al. (36) demonstrated that evening-type adults were more engaged with unhealthy dietary habits. In comparison, the effects of emotional states on nutrition, cognitive restrain, and inclinations to hunger were higher in individuals with morning-type chronotype (37). Similar to this study, the relationship between nutrition and emotion is higher in morning-type individuals than in other chronotypes in the present research.

Several studies have shown that individuals’ energy, carbohydrate and fat intakes in the evening chronotype were higher than those in the morning chronotype (18, 30, 38). In a study, it was shown that individuals with the evening chronotype tend to consume more energy and fat, especially in the evening hours (34). Contrary to these findings, in a review evaluating 35 studies investigating the relationship between chronotype and nutrition, it was concluded that the majority of them did not find a relationship between chronotypes and the energy intakes of individuals (36). In addition, it has been shown that the evening chronotype individuals consume less protein, calcium, magnesium, zinc, riboflavin, and vitamin B_6_ (18, 30, 35). In this study, significant differences were found between morning and evening chronotypes in terms of micronutrients, especially calcium and vitamin A. In other studies examining the relationship between food groups and chronotype, it was determined that individuals in the evening chronotype had less consumption of vegetables and fruits (39, 40) but more caffeine (41, 42) and alcohol consumption (38, 43). Low consumption of vegetables and fruits, which are sources of many micronutrients, and high consumption of caffeine and alcohol, which adversely affect the absorption of micronutrients, may cause this situation in the evening chronotype. Similarly, in this study, the fiber intake of individuals with the evening chronotype was lower than that of individuals with the morning chronotype. Moreover, in this study, one of the reasons why the differences in nutrients according to the chronotypes were not found to be statistically significant may be that BMI values did not differ according to different chronotypes. In addition, it is thought that in this study conducted during the COVID-19 period, efforts to improve the nutritional status of individuals to protect their health may be one of the reasons for the lack of difference in nutrient consumption. Since ASHN reflects more general eating habits, it is thought that there may be a relationship between chronotype and ASHN.

As a result of all these, as shown in this study, it turned out that the positive nutrition habits of the individuals in the evening chronotype were lower than those in the morning and intermediate chronotype (33). Inconsistency between sleep and wakefulness, hunger and satiety, light and dark cycles, and lower levels of healthy nutrition habits in the evening chronotype may reveal conditions that negatively affect glucose and lipid metabolism or blood pressure regulation in the chronic period (17). In the literature, it has been shown that the risk of developing diseases such as obesity (33) or metabolic syndrome (44, 45) increases, especially in individuals with the evening chronotype. From this point of view, it is one of the practices that may benefit nutrition professionals to intervene according to the chronotype that individuals tend to be in providing weight control and preventing these diseases.

This study had some limitations. First it was a cross-sectional study, so the present results could not determine the temporal direction of associations. It would have been more beneficial if the numbers of males and females were close to each other and the age range was more homogeneous. Thus, the difference between the genders could be better observed and the data could be better adapted to the general population. In evaluating diet quality, taking a three consecutive days dietary record instead of a 24 h dietary record may be a better indicator of reflecting general eating habits. Limitations of the study include not questioning other lifestyle factors of individuals, such as their physical activity status, with objective evaluation methods and not addressing whether they use a different method in sleep regulation.

## 5. Conclusion

This study determined the relationship between chronotype and healthy eating attitudes. According to the results of this study, a significant relationship was determined between chronotype and exhibiting healthy eating behavior. The ASHN scale used for this purpose is a predictor of MEQ. Moreover, this study confirmed that poor eating habits, which were also shown in other studies, are more common in the evening chronotype. In this way, nutrition professionals can see the necessity of taking precautions and regulations by considering the chronotypes of individuals in terms of gaining healthy eating behavior. In future studies, to better understand the effect of chronotype on nutrition, it will be useful to examine subjects such as chronotype and nutritional dependence, hunger and satiety periods, and nutritional content of meals.

## Data availability statement

The raw data supporting the conclusions of this article will be made available by the authors, without undue reservation.

## Ethics statement

The studies involving humans were approved by the Ethics Committee of the Gazi University. The studies were conducted in accordance with the local legislation and institutional requirements. The participants provided their written informed consent to participate in this study.

## Author contributions

HM: Conceptualization, Formal analysis, Investigation, Methodology, Writing – original draft, Writing – review & editing. BA: Conceptualization, Investigation, Supervision, Writing – original draft. SNV: Conceptualization, Investigation, Writing – original draft. SK: Investigation, Software, Writing – original draft. DA: Conceptualization, Writing – review & editing. SB: Supervision, Writing – review & editing.
